# A nomogram to predict postoperative new-onset cerebral infarction after revascularization of moyamoya disease in adults and its validation: a retrospective study

**DOI:** 10.3389/fneur.2025.1537755

**Published:** 2025-01-24

**Authors:** Zhen Wang, Jiacheng Yu, Yu Zhang, Jiaping Ruan, Xiaojie Liu, Sijia Ma, Jun Xie, Mimi Wu, Jinhua Bo, Yu’e Sun

**Affiliations:** ^1^Department of Anesthesiology, Nanjing Drum Tower Hospital, Clinical College of Nanjing Medical University, Nanjing, China; ^2^Department of Anesthesiology, Nanjing Drum Tower Hospital, The Affiliated Hospital of Nanjing University Medical School, Nanjing, China; ^3^Department of Anesthesiology, Chongqing General Hospital, Chongqing, China

**Keywords:** adult moyamoya disease, revascularization, postoperative new-onset cerebral infarction, nomogram, retrospective study

## Abstract

**Background:**

The new-onset cerebral infarction is frequent after revascularization of moyamoya disease (MMD) in adults, serving as a major public health issue worldwide. The present study aims to construct a nomogram to predict postoperative new-onset cerebral infarction (POCI) after revascularization of adult MMD.

**Materials and methods:**

Clinical data of 653 cases of adult MMD treated with revascularization were retrospectively analyzed. They were randomly divided into a training set (*n* = 457) and a validation set (*n* = 196) at a ratio of 7:3. Based on the risk factors of POCI after revascularization of adult MMD identified by logistic regression analysis and the corresponding regression coefficients, a nomogram was constructed. Its performance to predict POCI after revascularization of adult MMD was validated by calculating the area under the curve (AUC) and the decision curve analysis.

**Results:**

Univariate and multivariate logistic regression analyses showed that preoperative cerebral infarction (OR 2.548, 95% CI 1.357–4.787; *p* = 0.004), posterior cerebral artery anomalies (OR 2.106, 95% CI 1.157–3.834; *p* = 0.015), post-transit arterial development (OR 2.983, 95% CI 1.336–6.661; *p* = 0.008), pre-anesthesia mean arterial pressure > 102.830 mmHg (OR 3.329, 95% CI 1.938–5.721; *p* < 0.001), total operating time > 212.500 min (OR 2.256, 95% CI 1.239–4.140; *p* = 0.008), preoperative fibrinogen level > 2.750 g/L (OR 1.852, 95% CI 1.072–3.200; *p* = 0.027), and mean corpuscular hemoglobin concentration (OR 1.021, 95% CI 1.001–1.040; *p* = 0.038) were independent risk factors of POCI after revascularization of adult MMD. The AUC was 0.772 (95% CI 0.714–0.772) in the training set, and 0.718 (95% CI 0.603–0.833) in the validation set.

**Conclusion:**

Collectively, the newly established nomogram effectively and intuitively predicts the POCI after revascularization of adult MMD.

**Clinical trial registration:**

www.chictr.org, identifier ChiCTR2400087946.

## 1 Background

Moyamoya disease (MMD) is a progressive cerebrovascular disease characterized by stenosis of the cerebral arteries surrounding the circle of Willis and development of abnormal moyamoya vessels, which has become a leading cause of ischemic and hemorrhagic strokes (1, 2). Presently, revascularization is preferred to adult MMD that effectively halts disease progression and prevents future ischemic strokes (3). However, postoperative new-onset cerebral infarction (POCI) after revascularization remains high (4.0–33.0%) (4–7), resulting in a poor outcome, long length of stay and high economic costs.

Prior studies have identified several risk factors of postoperative cerebral ischemic complications of MMD and most of them cannot be modified (6–11). So far, the vascular condition of MMD is the mostly concerned predictive factor of POCI (5, 12). Besides the vascular condition, POCI is also closely linked with perioperative factors like preoperative comorbidities, intraoperative blood pressure (13), coagulation state, and surgical methods (8). A prediction model comprehensively incorporating the above-mentioned factors is urgently needed to visualize the possibility of POCI after revascularization of adult MMD, thus providing survival benefits to affected people.

In the present study, we comprehensively analyzed clinical data of POCI in adult MMD patients treated with revascularization, and constructed a nomogram. Its performance was later validated, aiming to provide a useful tool to screen high-risk population of POCI.

## 2 Methods and materials

### 2.1 Study population

This was a single-center, large-scale retrospective observational cohort study with the approval of the institutional review board (No. 2023-455-01) on October 9, 2023 and waiver of informed consent. It was registered at www.chictr.org (No. ChiCTR2400087946) on August 7, 2024. Elective intracranial extracranial vascular bypass between February 2018 and February 2024 at Nanjing Drum Tower Hospital were retrospectively screened. The exclusion criteria were as follows: pediatric MMD, geriatric MMD, those lacking magnetic resonance angiography, digital subtraction angiography, or other preoperative and postoperative data, and individuals with cerebrovascular lesions caused by atherosclerosis, autoimmune disease, meningitis, brain tumors, Down syndrome, von Recklinghausen’s disease, head injury, or head irradiation (14).

### 2.2 Anesthesia management

A standardized anesthesia protocol was established in our center to ensure a homogeneous management during the induction and maintenance of anesthesia, and regulatory phases of blood pressure, arterial blood carbon dioxide partial pressure, and fluid therapy. Detailed protocols during the anesthesia management were described in Supplementary Table S1.

### 2.3 Definition of POCI

**Figure 1:**
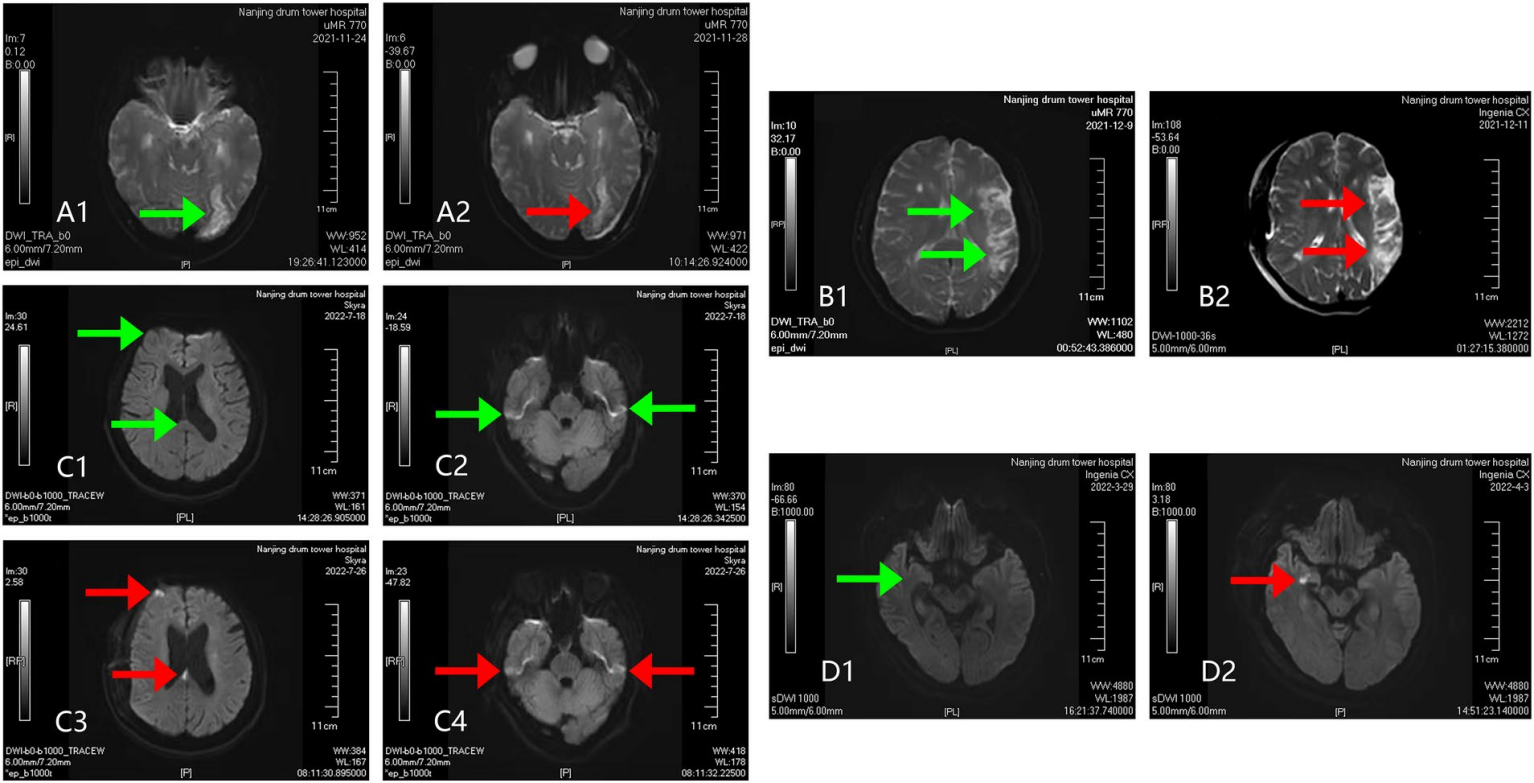
Representative imaging scans of 4 cases of POCI. In Case A classified as POCI (−), there lacked obvious manifestations of cerebral infarction before **(A1)** or after surgery **(A2)**, only showing intracranial softening foci. In Case B classified as POCI (−), a preoperative subacute cerebral infarction **(B1)** without postoperative progression **(B2)** was visualized on imaging scans. In Case C classified as POCI (+), no obvious infarction foci were preoperatively observed in the corpus callosum **(C1)** or the right frontoparietal lobe **(C2)**. Postoperatively, new acute infarction was identified in the corpus callosum **(C3)**, while the preoperatively identified infarct foci on the right side remained unchanged **(C4)**. In Case D classified as POCI (+), there lacked obvious manifestations of cerebral infarction preoperatively **(D1)**, but presented a new-onset acute cerebral infarction in the left hemi-oval center postoperatively **(D2)**.

POCI was defined as newly detected cerebral infarctions on imaging scans within 7 days of surgery compared to preoperative assessments. New-onset cerebral infarctions were low-density foci visualized on postoperative electron computed tomography (CT) scans or high-signal areas on magnetic resonance diffusion-weighted imaging sequences that were not associated with ischemic symptoms. New-onset cerebral hemorrhage must be excluded (5, 15). Representative imaging scans before and after surgery were shown in Figure 1.

### 2.4 Data collection and processing

Clinical data were available from the medical record system and anesthesia management system. All biochemical indices were obtained within 4 days preoperatively and independently verified by two investigators. Missing values were addressed using the list deletion method.

#### 2.4.1 Preoperative indicators

Briefly, three categories of preoperative data were recorded. First, baseline and demographic data, including gender, age, body mass index, the American Society of Anesthesiologists physical status classification, preoperative history and comorbid symptoms (e.g., comorbid hypokinesia, speech dysfunction, visual impairment and loss of consciousness), preoperative medications (e.g., antihypertensive agents, hypoglycemic agents, aspirin, clopidogrel), and preoperative comorbidities (e.g., hypertension, diabetes mellitus, cerebral infarction, stroke, cerebral hemorrhage, intracranial aneurysm, brain atrophy). Second, preoperative laboratory testing, including complete blood count (CBC), liver and kidney function, coagulation function, and biochemical testing. Third, the severity and vascular involvement of MMD, including the type of MMD (hemorrhagic, ischemic, and asymptomatic), and vascular involvement (involvement of the anterior cerebral arteries [ACA], middle cerebral arteries [MCA], internal carotid arteries, posterior cerebral arteries [PCA], vertebral basilar arteries, and opening of the anterior and posterior traffic arteries).

#### 2.4.2 Surgery-related and anesthesia-related indicators

Revascularization was performed by the same chief surgeon. The following surgery-related indicators were recorded: surgical approach (direct, indirect, and combined revascularization), number of surgeries (first or second revascularization), side of the surgery (left or right), recipient vessel for revascularization (posterior branch, anterior branch, apical branch and frontal branch of the superficial temporal artery), and the total operating time (TOT).

The following anesthesia-related indicators were recorded: preoperative noninvasive blood pressure (systolic blood pressure [SBP], diastolic blood pressure [DBP], and mean arterial pressure [MAP]), total anesthesia time (TAT), fluid intake and output (total outflow, total inflow, hemorrhage, colloid replenishment, and crystalloid replenishment), total consumption of analgesic medication, and maximum and minimum values of end-expiratory carbon dioxide.

#### 2.4.3 Conversion of continuous variables into dichotomous variables

Most of clinical data in this study were continuous variables. To enhance the clinical applicability, continuous variables were converted into dichotomous variables using specific cutoff values for subsequent statistical analyses. The optimal cutoff values were selected by referencing normal physiological values provided by our laboratory center, or constructing a receiver operating characteristic (ROC) curve to calculate the Youden index. Detailed cutoff values were listed in Supplementary Table S2.

### 2.5 Statistical analysis

#### 2.5.1 Analytical methods

Descriptive analysis and binary logistic regression analyses were performed by SPSS 27 (IBM, Armonk, NY, United States), and other statistical analyses were performed by R software (version 4.4.0, http://www.R-project.org). The data was randomly divided into training and validation sets at a ratio of 7:3. The training set was used for feature selection and model building, while the validation set was used to evaluate the effect of the trained model. Categorized variables were described by the frequency and percentage; continuous variables obeying normal distribution were described as mean ± standard deviation; and skewed distribution data were described as median and interquartile range (P25, P75). Chi-square test for categorical data, t-test for continuous variables obeying normal distribution, and Mann–Whitney U-test for continuous variables not obeying normal distribution were performed. All tests were two-tailed and *p* < 0.05 was defined as statistically significant.

#### 2.5.2 Construction of a nomogram and its validation

Initially, stepwise backward regression was performed to identify variables significantly associated with POCI in the training set (*p* < 0.05). Those with a significant difference were further subjected to the multivariable logistic regression. Then, a nomogram was constructed to predict POCI after revascularization of adult MMD. Its performances, including the discrimination, calibration, and clinical applicability were tested by ROC, calibration curve, decision curve analysis (DCA), respectively. The generalization of the nomogram was finally validated in the validation set. In details, an AUC > 0.70 indicated the acceptable discrimination of the nomogram. The calibration was evaluated by comparing predicted values with observed results, visualized by a calibration curve plot using a 1,000 bootstrap resampling procedure. DCA was used to quantify the net benefits at different threshold probabilities (16).

## 3 Results

### 3.1 Baseline characteristics

**Figure 2:**
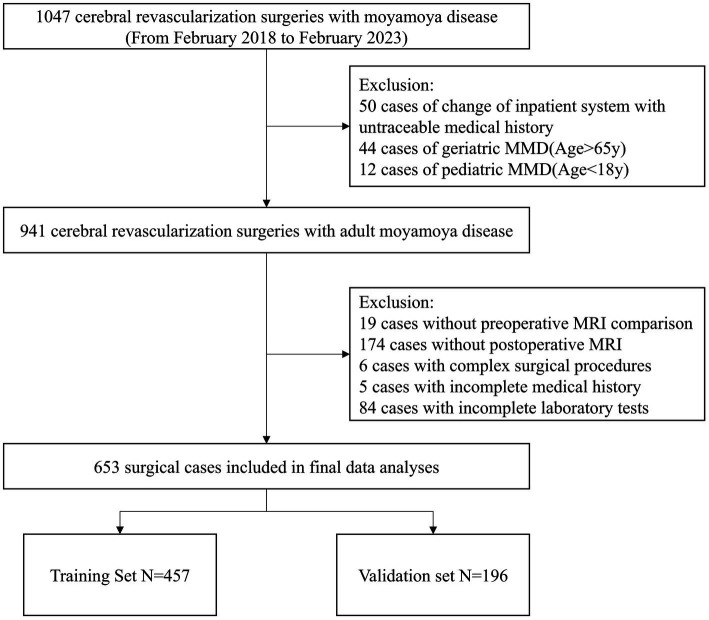
Flow chart of the study.

A total of 1,047 cases of MMD were initially screened. After excluding 50 cases lacking medical history, 44 cases of geriatric MMD, and 12 cases of pediatric MMD, 941 cases were saved. After review of the perioperative data, 288 cases were removed. Thus, a total of 653 cases were enrolled in the study. The included cases were randomly divided into a training set and a validation set on a 7:3 basis, involving 457 cases in the training set and 196 cases in the validation set (Figure 2).

Of the included cases, 308 (47.17%) were male and 345 (52.83%) were female patients, with a mean age of 47.90 ± 9.48 years. POCI confirmed by positive imaging manifestations was found in 102 (15.62%) cases, consisting of 48 (7.35%) cases of asymptomatic cerebral infarction and 54 (8.27%) of symptomatic cerebral infarction. Due to the randomized grouping, the incidence of POCI was comparable between the training and validation sets (*p* > 0.05). Demographic data between the training set and validation set were detailed in Table 1.

**Table 1:** General patient characteristics (*n* = 653).

Variables	Total (*n* = 653)	Validation set (*n* = 196)	Training set (*n* = 457)	*t*/χ^2^	*p*
Age (years)	47.90 ± 9.48	48.01 ± 9.00	47.85 ± 9.69	0.19	0.847*
BMI (kg/m^2^)	24.95 ± 3.28	24.80 ± 3.00	25.01 ± 3.39	−0.76	0.448*
SBP on admission (mmHg)	130.99 ± 14.91	132.29 ± 14.74	130.43 ± 14.96	1.46	0.145*
DBP on admission (mmHg)	79.43 ± 10.40	79.84 ± 10.37	79.26 ± 10.42	0.65	0.513*
TOT (min)	227.65 ± 51.29	227.97 ± 51.06	227.51 ± 51.45	0.10	0.917*
TAT (min)	268.47 ± 55.76	269.36 ± 55.58	268.09 ± 55.89	0.27	0.790*
ASA (*n*, %)				0.67	0.717
II	41 (6.28)	12 (6.12)	29 (6.35)		
III	582 (89.13)	173 (88.27)	409 (89.50)		
IV	30 (4.59)	11 (5.61)	19 (4.16)		
Sex (*n*, %)				2.14	0.144
Male	308 (47.17)	101 (51.53)	207 (45.30)		
Female	345 (52.83)	95 (48.47)	250 (54.70)		
POCI (*n*, %)				0.01	0.928
Negative	551 (84.38)	165 (84.18)	386 (84.46)		
Positive	102 (15.62)	31 (15.82)	71 (15.54)		
Type of POCI (*n*, %)				3.65	0.161
Negative	551 (84.38)	165 (84.18)	386 (84.46)		
Asymptomatic	48 (7.35)	19 (9.69)	29 (6.35)		
Symptomatic	54 (8.27)	12 (6.12)	42 (9.19)		
Types of MMD (*n*, %)				0.59	0.743
Asymptomatic	156 (23.89)	47 (23.98)	109 (23.85)		
Infarcted	326 (49.92)	94 (47.96)	232 (50.77)		
Hemorrhagic	171 (26.19)	55 (28.06)	116 (25.38)		
Side of injury (*n*, %)				0.03	0.871
Left	303 (46.40)	90 (45.92)	213 (46.61)		
Right	350 (53.60)	106 (54.08)	244 (53.39)		
EMS (*n*, %)				0.01	0.908
Negative	538 (82.39)	162 (82.65)	376 (82.28)		
Positive	115 (17.61)	34 (17.35)	81 (17.72)		
Recipient vessel (*n*, %)				3.25	0.517
No revascularization	50 (7.66)	19 (9.69)	31 (6.78)		
STA-AB	95 (14.55)	26 (13.27)	69 (15.10)		
STA-PB	437 (66.92)	126 (64.29)	311 (68.05)		
STA-ApB	69 (10.57)	24 (12.24)	45 (9.85)		
STA-FB	2 (0.31)	1 (0.51)	1 (0.22)		
Types of surgery (*n*, %)				1.78	0.411
Direct	146 (22.36)	41 (20.92)	105 (22.98)		
Indirect	53 (8.12)	20 (10.20)	33 (7.22)		
Combined	454 (69.53)	135 (68.88)	319 (69.80)		
Number of surgeries (*n*, %)				0.06	0.808
First	474 (72.59)	141 (71.94)	333 (72.87)		
Second	179 (27.41)	55 (28.06)	124 (27.13)		

BMI, body mass index; SBP, systolic blood pressure; DBP, diastolic blood pressure; TOT, total operating time; TAT, total anesthesia time; ASA, the American Society of Anesthesiology; EMS, Encephalo-Myo-Synangiosis; STA-AB, anterior branch of the superficial temporal artery; STA-PB, posterior branch of the superficial temporal artery; STA-ApB, apical branch of the superficial temporal artery; STA-FB, frontal branch of the superficial temporal artery. The symbol <*> denotes that the associated variable is continuous. Conversely, variables without this marking are categorical.

### 3.2 Baseline characteristics of the training set

Of the 457 cases of adult MMD in the training set, 207 (45.30%) were male and 250 (54.70%) were female patients, with a mean age of 47.80 ± 9.77 years. Divided by the presence of POCI, there were significant differences in the SBP, DBP and mean corpuscular hemoglobin concentration (MCHC) on admission, type of MMD, and occurrence of cerebral hemorrhage (*p* < 0.05, Table 2).

**Table 2:** Baseline characteristics of patients in the training set (*n* = 457).

Variables	POCI negative (*n* = 386)	POCI positive (*n* = 71)	t/χ^2^/U	*p*
Age (years)	47.80 ± 9.77	48.14 ± 9.30	−0.28^a^	0.783
BMI (kg/m^2^)	24.57 (22.49, 26.80)	24.33 (22.91, 26.56)	−0.003^b^	0.997
SBP on admission (mmHg)	131 (120, 140)	140 (125, 150)	−4.062^b^	**<0.001**
DBP on admission (mmHg)	78 (71, 86)	90 (75, 92)	−3.702^b^	**<0.001**
TOT (min)	220 (190, 255)	240 (207, 265)	−1.861^b^	0.063
TAT (min)	260 (230, 295)	278 (250, 265)	−1.977^b^	0.048
WBC (10^9^/L)	5.7 (4.8, 6.9)	5.8 (5.15, 7.0)	−1.365^b^	0.172
Neutrophil percentage (%)	58.93 ± 8.29	57.53 ± 7.25	1.34^a^	0.182
Lymphocyte percentage (%)	31.81 ± 7.85	33.08 ± 6.92	−1.27^a^	0.204
HB (g/L)	136 (124, 147)	140 (128, 147)	−1.301^b^	0.172
MCHC (g/L)	335 (326, 343)	338 (330, 346)	−2.325^b^	**0.020**
PLT (10^9^/L)	208 (171, 248)	219.5 (173, 253)	−0.447^b^	0.655
TBIL (μmol/L)	9.7 (6.7, 13.0)	9.9 (7.8, 12.5)	−0.516^b^	0.606
Alb (g/L)	42.1 (40.6, 43.9)	42.3 (40.8, 43.6)	−0.207^b^	0.836
ASA (*n*, %)			–	0.444
II	27 (6.99)	2 (2.82)		
III	342 (88.60)	67 (94.37)		
IV	17 (4.40)	2 (2.82)		
Sex (*n*, %)			3.15^c^	0.076
Male	168 (43.52)	39 (54.93)		
Female	218 (56.48)	32 (45.07)		
Types of MMD (*n*, %)			15.65^c^	**<0.001**
Asymptomatic	97 (25.13)	12 (16.90)		
Infarcted	181 (46.89)	51 (71.83)		
Hemorrhagic	108 (27.98)	8 (11.27)		
Hypertension (*n*, %)			2.56^c^	0.278
No hypertension	222 (57.51)	37 (52.11)		
High-risk type	73 (18.91)	11 (15.49)		
Very high-risk type	91 (23.58)	23 (32.39)		
Diabetes mellitus (*n*, %)			1.61^c^	0.204
No	322 (83.42)	54 (77.14)		
Yes	64 (16.58)	16 (22.86)		
Cerebral hemorrhage (*n*, %)			10.46^c^	**0.001**
No	278 (72.02)	64 (90.14)		
Yes	108 (27.98)	7 (9.86)		
History of intracranial aneurysm (*n*, %)			2.01^c^	0.156
No	360 (93.51)	70 (98.59)		
Yes	25 (6.49)	1 (1.41)		
Encephalanalosis (*n*, %)			0.00^c^	0.983
No	295 (77.02)	54 (77.14)		
Yes	88 (22.98)	16 (22.86)		
Side of injury (*n*, %)			0.00^c^	0.981
Right	180 (46.63)	33 (46.48)		
Left	206 (53.37)	38 (53.52)		
ESM (*n*, %),			1.33^c^	0.248
No	321 (83.16)	55 (77.46)		
Yes	65 (16.84)	16 (22.54)		
Recipient vessel (*n*, %)			-	0.329
No revascularization	27 (6.99)	4 (5.63)		
STA-AB	57 (14.77)	12 (16.90)		
STA-PB	262 (67.88)	49 (69.01)		
STA-PaB	40 (10.36)	5 (7.04)		
STA-FB	0 (0.00)	1 (1.41)		
Types of surgery (*n*, %)			3.14^c^	0.208
Direct	83 (21.50)	22 (30.99)		
Indirect	29 (7.51)	4 (5.63)		
Combined	274 (70.98)	45 (63.38)		
Number of surgeries (*n*, %)			0.43^c^	0.511
First	279 (72.28)	54 (76.06)		
Second	107 (27.72)	17 (23.94)		

a *T-test.*

b Mann–Whitney U test.

c Chi-square test.

BMI, body mass index; SBP, systolic blood pressure; DBP, diastolic blood pressure; TOT, total operating time; TAT, total anesthesia time; WBC, white blood cell count; HB, hemoglobin; MCHC, mean corpuscular hemoglobin concentration; PLT, platelet count; TBIL, total bilirubin; Alb, albumin; ASA, the American Society of Anesthesiology; EMS, Encephalo-Myo-Synangiosis; STA-AB, anterior branch of the superficial temporal artery; STA-PB, posterior branch of the superficial temporal artery; STA-ApB, apical branch of the superficial temporal artery; STA-FB, frontal branch of the superficial temporal artery. The values in bold indicate statistical significance at the *P* < 0.05 level.

### 3.3 Univariate and logistic regression analyses of POCI

Univariate logistic regression initially identified 14 variables significantly correlated with POCI in adult MMD patients after revascularization (Supplementary Table S3), including the type of MMD (OR 3.218, 95% CI 1.523–6.798; *p* = 0.002), history of cerebral infarction (OR 3.152, 95% CI 1.752–5.670; *p* < 0.001), PCA anomalies (OR 2.484, 95% CI 1.442–2.272; *p* = 0.001), post-transit arterial development (PTAD, OR 2.250, 95% CI 1.120–4.524; *p* = 0.023), pre-anesthesia MAP>102.83 mmHg (OR 3.733, 95% CI 2.244–6.209; *p* < 0.001), pre-anesthesia SBP > 140 mmHg (OR 2.848, 95% CI 1.720–4.718; *p* < 0.001), pre-anesthesia DBP > 85.5 mmHg (OR 2.810, 95% CI 1.697–4.653; *p* < 0.001), TOT>212.5 min (OR 2.848, 95% CI 1.720–4.718; *p* = 0.026), TAT > 245 min (OR 2.156, 95% CI 1.180–3.940; *p* = 0.013), MCHC (OR 1.021, 95% CI 1.003–1.040; *p* = 0.002), alkaline phosphatase (OR 1.013, 95% CI 1.002–1.024; *p* = 0.022), direct bilirubin (OR 1.220, 95% CI 1.035–1.437; *p* = 0.017), fibrinogen (FIB) >2.75 g/L (OR 1.982, 95% CI 1.202–3.266; *p* = 0.007), and history of cerebral hemorrhage (OR 0.486, 95% CI 0.252–0.937; *p* = 0.031) (Table 3).

**Table 3:** Independent risk factors of POCI identified by univariate and stepwise regression multivariate analyses.

	Univariate analysis		Multivariate analysis	
Variables	OR (95% CI)	*p* value	OR (95% CI)	*p* value
Sex				
Male	Reference			
Female	0.732 (0.447–1.2)	0.216		
Age (years)				
18–39	Reference			
40–59	1.351 (0.778–2.344)	0.285		
60–65	0.75 (0.207–2.714)	0.661		
BMI (kg/m^2^)				
Low-weight (<18.5)	Reference			
Normal-weight (18.5–24.9)	0.346 (0.03–3.941)	0.392		
Overweight (25–30)	0.440 (0.039–4.992)	0.508		
Obese (>30)	0.414 (0.035–4.939)	0.486		
ASA				
II	Reference			
III	2.670 (0.619–11.511)	0.188		
IV	3.421 (0.599–19.555)	0.167		
Types of MMD				
Infarcted	Reference			
Hemorrhagic	3.218 (1.523–6.798)	0.002		
Asymptomatic	1.308 (0.535–3.197)	0.556		
Clinical symptoms				
Hypokinesia				
No	Reference			
Yes	1.285 (0.776–2.126)	0.330		
Speech disorders				
No	Reference			
Yes	1.644 (0.853–3.171)	0.138		
Visual impairment				
No	Reference			
Yes	1.729 (0.663–4.509)	0.263		
Loss of consciousness				
No	Reference			
Yes	0.315 (0.095–1.041)	0.058		
Complications of MMD				
Cerebral infarction				
No	Reference		Reference	
Yes	3.152 (1.752–5.670)	<0.001	2.548 (1.357–4.787)	0.004
Cerebral hemorrhage				
No	Reference			
Yes	0.486 (0.252–0.937)	0.031		
Stroke				
No	Reference			
Yes	1.623 (0.990–2.662)	0.055		
Intracranial aneurysms				
No	Reference			
Yes	0.174 (0.023–1.303)	0.089		
EMS				
No	Reference			
Yes	1.120 (0.626–2.006)	0.702		
Comorbidities				
Hypertension				
No hypertension	Reference			
High-risk type	0.611 (0.293–1.271)	0.187		
Very high-risk type	1.250 (0.707–2.209)	0.443		
Diabetes mellitus				
No	Reference			
Yes	1.151 (0.617–2.147)	0.659		
Medication history				
Antihypertensive drugs				
No	Reference			
Yes	1.085 (0.655–1.798)	0.750		
Antihyperglycemic drugs				
No	Reference			
Yes	1.140 (0.601–2.161)	0.689		
Aspirin				
No	Reference			
Yes	1.639 (0.932–2.881)	0.086		
Clopidogrel				
No	Reference			
Yes	1.869 (0.579–6.032)	0.296		
Cerebrovascular conditions				
ACA				
No	Reference			
Yes	1.131 (0.636–2.013)	0.675		
ICA				
No	Reference			
Yes	1.135 (0.67–1.925)	0.637		
MCA				
No	Reference			
Yes	0.396 (0.035–4.421)	0.452		
PCA				
No	Reference		Reference	
Yes	2.482 (1.442–4.272)	0.001	2.106 (1.157–3.834)	0.015
VBA				
No	Reference			
Yes	1.275 (0.503–3.233)	0.609		
Former transportation arterial development				
No	Reference			
Yes	1.830 (0.697–4.805)	0.220		
PTAD				
No	Reference		Reference	
Yes	2.250 (1.120–4.524)	0.023	2.983 (1.336–6.661)	0.008
Surgery-related indicators				
Side of injury				
Left	Reference			
Right	1.038 (0.633–1.704)	0.882		
Number of surgeries				
First	Reference			
Second	0.917 (0.53–1.588)	0.757		
Types of surgery				
Direct	Reference			
Indirect	0.364 (0.101–1.308)	0.122		
Combined	0.722 (0.410–1.270)	0.258		
Use of edaravone				
No	Reference			
Yes	1.495 (0.858–2.607)	0.156		
Perioperative indicators				
SBP				
<140 mmHg	Reference			
≥140 mmHg	2.848 (1.720–4.718)	<0.001		
DBP				
<85.5 mmHg	Reference			
≥85.5 mmHg	2.810 (1.697–4.653)	<0.001		
MAP				
<102.83 mmHg	Reference		Reference	
≥102.83 mmHg	3.733 (2.244–6.209)-	<0.001	3.329 (1.938–5.721)	<0.001
TOT				
<212.5 min	Reference		Reference	
≥212.5 min	1.856 (1.079–3.182)	0.026	2.265 (1.239–4.140)	0.008
TAT				
<245 min	Reference			
≥245 min	2.156 (1.180–3.940)	0.013		
Laboratory indicators				
WBC (10^9^/L)	1.081 (0.932–1.253)	0.302		
NLR	0.905 (0.683–1.200)	0.488		
HB (g/L)				
≥130	Reference			
<130	0.816 (0.484–1.374)	0.444		
Mean Hb (p g)	1.065 (0.982–1.156)	0.130		
MCHC (g/L)	1.021 (1.003–1.040)	0.020	1.021 (1.001–1.040)	0.038
RDW (%) PLT (10^9^/L)	0.842 (0.663–1.068)	0.157		
≥125	Reference			
<125	1.445 (0.601–3.472)	0.411		
TP (g/dL)	1.008 (0.958–1.060)	0.754		
Alb				
≥40 g/L	Reference			
<40 g/L	1.022 (0.531–1.967)	0.948		
A/G	0.743 (0.263–2.099)	0.574		
LDH (U/L)	1.003 (0.996–1.010)	0.350		
ALP (U/L)	1.013 (1.002–1.024)	0.022		
TBIL (μmol/L)	1.019 (0.974–1.067)	0.402		
DBIL (μmol/L)	1.220 (1.035–1.437)	0.017		
CRP (mg/L)	0.991 (0.923–1.064)	0.810		
TC (mmol/L)	0.773 (0.594–1.057)	0.054		
TG (mmol/L)	0.917 (0.699–1.202)	0.530		
HDL (mmol/L)	0.806 (0.348–1.867)	0.614		
LDL (mmol/L)	0.726 (0.527–1.001)	0.051		
FC (mmol/L)	0.857 (0.435–1.687)	0.655		
FBG (mmol/L)	1.092 (0.943–1.264)	0.238		
P_V_CO_2_				
≥24.65 mmol/L	Reference			
<24.65 mmol/L	0.608 (0.353–1.047)	0.073		
PT(s)	0.951 (0.746–1.212)	0.684		
APTT(s)	0.957 (0.855–1.072)	0.448		
TT(s)	0.996 (0.775–1.281)	0.978		
INR	0.450 (0.024–8.331)	0.592		
FIB				
<2.75 g/L	Reference		Reference	
≥2.75 g/L	1.982 (1.202–3.266)	0.007	1.852 (1.072–3.200)	0.027

POCI, postoperative new-onset cerebral infarction; OR, odds ratio; CI, confidence interval; BMI, body mass index; ASA, the American Society of Anesthesiology; MMD, moyamoya disease; EMS, Encephalo-Myo-Synangiosis; ACA, anterior cerebral arteries; MCA, middle cerebral arteries; ICA, internal carotid arteries; PCA, posterior cerebral arteries; VBA, vertebral basilar arteries; PTAD, post-transit arterial development; SBP, systolic blood pressure; DBP, diastolic blood pressure; MAP, mean arterial pressure; TOT, total operating time; TAT, total anesthesia time; WBC, white blood cell count; NLR, neutrophil-to-lymphocyte ratio; HB, hemoglobin; MCHC, mean corpuscular hemoglobin concentration; RDW, red cell distribution width; PLT, platelet count; TP, total protein; Alb, albumin; A/G, albumin-to-globulin ratio; LDH, lactate dehydrogenase; ALP, alkaline phosphatase; TBIL, total bilirubin; DBIL, direct bilirubin; CRP, C-reactive protein; TC, total cholesterol; TG, triglyceride; HDL, high-density lipoprotein; LDL, low-density lipoprotein; FC, free cholesterol; FBG, fasting blood glucose; P_V_CO_2_, venous blood carbon dioxide; PT, prothrombin time; APTT, activated partial thromboplastin time; INR, international normalized ratio; FIB, fibrinogen.

Finally, 7 independent risk factors of POCI were screened by stepwise backward regression analysis, including the history of cerebral infarction (OR 2.548, 95% CI 1.357–4.787; *p* = 0.004), PCA anomalies (OR 2.106, 95% CI 1.157–3.834; *p* = 0.015), PTAD (OR 2.983, 95% CI 1.336–6.661; *p* = 0.008), pre-anesthesia MAP>102.83 mmHg (OR 3.329, 95% CI 1.938–5.721; *p* < 0.001), TOT>212.5 min (OR 2.265, 95% CI 1.239–4.140; *p* = 0.008), MCHC (OR 1.021, 95% CI 1.001–1.040; *p* = 0.038), and preoperative FIB>2.75 g/L (OR 1.852, 95% CI 1.072–3.200; *p* = 0.027) (Table 3).

### 3.4 A nomogram to predict POCI after revascularization of adult MMD

**Figure 3:**
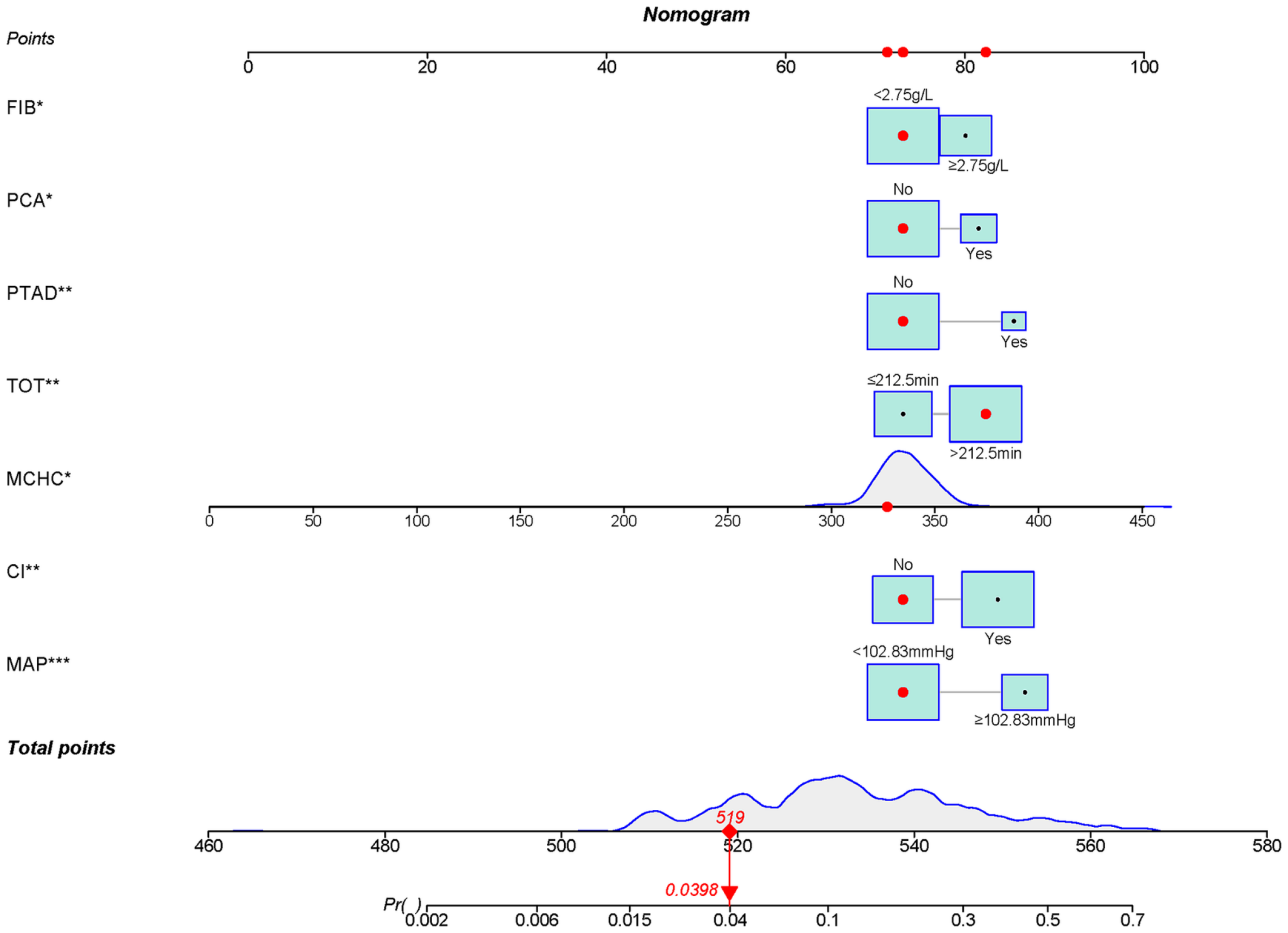
A nomogram to predict POCI after revascularization of adult MMD. In the nomogram, parameters marked with an asterisk (*) indicate that they remain statistically significant in the final model, whereas those without an asterisk indicate that they are not statistically significant. For continuous variables, the distribution is represented as a frequency distribution curve; for categorical variables, the distribution is depicted using green squares, where larger squares represent higher frequencies of individuals at the corresponding level. Additionally, the first observation in the dataset is highlighted on the graph with a red dot, and its corresponding points are also indicated by red dots.

Incorporating the 7 independent risk factors of POCI, we constructed a nomogram (Figure 3) and the corresponding risk score of each variable was listed in Supplementary Table S4. By calculating the total score of the 7 parameters, a higher score predicted a greater possibility of POCI after revascularization of adult MMD.

For example, a male adult patient with MMD who FIB<2.75 g/L, PCA = No, PATD = No, TOT>212.5 min, MCHC =325, CI = No, MAP <102.83 mmHg, and was graded with 73, 73, 73, 82, 72, 73 and 73 points, respectively. A total score of 519 points in this nomogram indicated a 3.98% probability of POCI. In addition, we constructed a web-based online dynamic nomogram to estimate the probability of new-onset cerebral infarction after revascularization of moyamoya disease, which can be accessed at https://predict-calculate.shinyapps.io/DynNomapp/.

**Figure 4:**
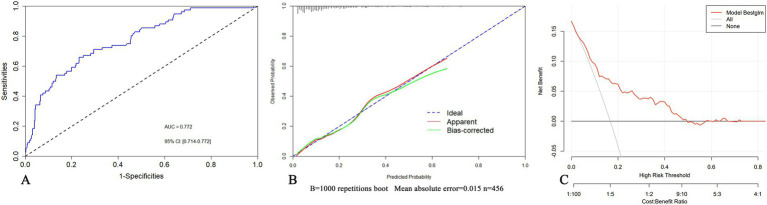
The validation of the nomogram in the training set by ROC curves **(A)**, calibration curves **(B)** and DCA **(C)**.

Validated in the training set, the AUC was 0.772 (95% CI 0.714–0.772) (Figure 4A). The calibration curves showed bias-correction and an apparent curve similar to the ideal line (Figure 4B), demonstrating a good agreement between the predicted and observed extent of POCI. The DCA curve further demonstrated the gain of net benefit at a threshold of 3–60% (Figure 4C).

### 3.5 Internal validation of the nomogram

**Figure 5:**
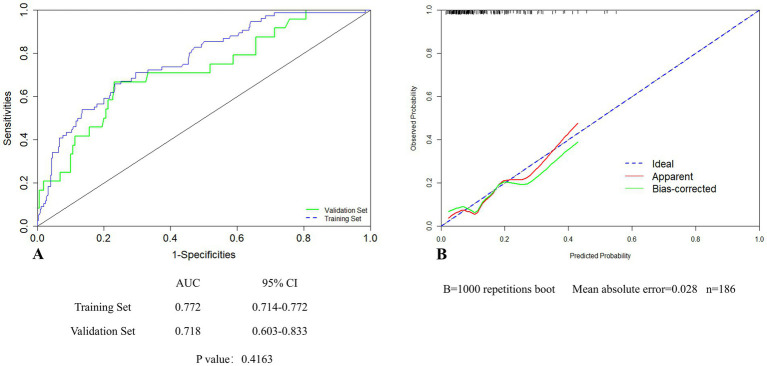
Internal validation of the nomogram in the validation set by ROC **(A)** and calibration curves **(B)**.

In the validation set, the AUC of the nomogram was 0.718 (95% CI: 0.603–0.833), showing no significant difference in comparison to that of the training set (Figure 5A). For the calibration curves, the apparent curves approached to the ideal curves were indicative of a good performance of prediction (Figure 5B). Overall, the nomogram exhibited good discrimination, calibration, and clinical applicability.

## 4 Discussion

Through thoroughly analyzed perioperative data of adult MMD patients treated with revascularization, including the generation information, demographic data, laboratory testing, imaging findings, surgery-related indicators and anesthesia-related indicators, we found that history of cerebral infarction, PCA anomalies, PTAD, pre-anesthesia MAP>102.83 mmHg, TOT>212.5 min, MCHC, and preoperative FIB>2.75 g/L were significantly correlated with POCI. In addition, a nomogram based on these factors was established to identify high-risk patients of POCI.

The incidence of POCI in our study was 15.62%, and that of symptomatic cerebral infarction was 8.27%. In line with our study, a multicenter retrospective study reported an ischemic stroke incidence of 15.6% within 30 days after revascularization (17). In addition, William et al. (18) reported a 30-day incidence of postoperative stroke in MMD patients at 14.4%. Given the high incidence of POCI in MMD patients, early identification of the high-risk population is of great importance to favor the individualized treatment and long-term prognosis.

Through analyzing baseline and demographic data of participants, especially the vascular conditions associated with MMD, we found that history of cerebral infarction, PCA anomalies and PTAD were significantly correlated with POCI. Accordingly, preoperative cerebral infarction was reported to be an indicator for instability of regional cerebral blood flow (rCBF) and have inadequate collateralization pathways to compensate for hemodynamic impairment (19–21), thus resulting in postoperative ischemic complications. Besides, the involvement of abnormal collateral vessels as a compensatory mechanism is important in MMD patients at a high risk of POCI. PCA anomalies (e.g., stenosis, occlusion, formation of collateral vessels), either in the main trunk or its branches, threaten the risk of cerebral infarction. Notably, smoky vessels in the PCA are associated with higher Suzuki stages and higher probability of POCI in MMD patients (5). Furthermore, meningeal collateral branches arising from the PCA provide the collateral blood flow in patients with advanced MMD. Individuals with the involvement of PCA were reported to be more vulnerable to the hemodynamic stress of general anesthesia and surgical revascularization (7). Although the rCBF and perfusion in the operated hemisphere with the involved PCA can be preserved or even enhanced by a direct revascularization, those in the contralateral hemisphere often remain inadequate and thus result in POCI (22). Posterior circulatory compensation typically refers to the supply of blood from the PCA to the original donor areas of MCA and ACA through various collateral and anastomotic branches (5). In our study, there was an increased probability of POCI in adult MMD patients with PTAD, which might be attributed to more severe Suzuki’s angiographic stage of MMD and more reduced tolerance to cerebral ischemia. The resulted severe hemodynamic failure can lead to the ischemic emergence (8, 12).

Our results showed that MCHC and FIB>2.75 g/L were independent risk factors of POCI after revascularization of adult MMD. These results are similar to those of a previous clinical trial conducted by Bao et al. (23) who found that populations residing in plateau regions experience increased blood viscosity due to heightened metabolic activity in the brain and a greater reliance on oxygen, leading to elevated risks of vasospasm, thrombosis and infarction. We consistently identified a significant correlation of high-level MCHC with increased risk of POCI. However, MCHC could be influenced by confounding factors like sampling, lipid levels, hemolysis, erythrocyte agglutination, and spherocytes, which should be adjusted in future prospective studies (24). In addition, elevated FIB was associated with increased risks of cardiovascular diseases like coronary heart disease and stroke (25). Serving as a critical influencing factor of coagulation cascade, an increase in FIB indicates a hypercoagulable state that impairs endothelial function, reduces blood flow and forms thrombosis. Previous findings have illustrated the correlation of high FIB with Alzheimer’s disease and vascular dementia, rather than CRP (26), which are consistent with our findings and further highlight the vascular property of FIB involved in POCI.

Surgery also linked with POCI in adult patients with MMD. A meta-analysis suggested that combined bypass and direct bypass offer significant benefits to the outcomes of advanced stroke and cerebral hemorrhage (27). Conversely, a retrospective multicenter study did not identify significant differences in the recurrence of stroke, perioperative stroke, and mortality between MMD patients treated with indirect and direct bypass (or combined bypass) (17). In our study, surgical procedures did not influence the incidence of POCI. In addition, we found that the incidence of POCI was significantly higher in MMD patients with prolonged TOT, which may be attributed to the more severe ischemia in the affected vessel that greatly challenged the surgical procedures of intraoperative bypass, such as the management of massive hemostasis, selection of the recipient vessel, and prevention of recurrent local bleeding (28).

Anesthesia is a crucial event involved in the surgical treatment of MMD. Our findings indicated that MAP on admission higher than 102.83 mmHg was a significant predictor of POCI in adult MMD. Effective perioperative anesthesia management of blood pressure and carbon dioxide partial pressure is the cornerstone of few postoperative complications and mortality. Our data showed that elevated SBP, DBP and MAP on admission were significantly correlated with POCI, which were consistent with previous findings (13). Due to preoperative ischemia, an elevated peripheral blood pressure is essential to maintain cerebral perfusion. MMD patients at advanced stage experienced greater ischemia, indirectly requiring the more pronounced increase in the preoperative MAP. The use of antihypertensive agents during the perioperative period to reduce intraoperative hemorrhage and expand the surgical field may further decrease cerebral perfusion, exacerbate ischemia and cause cerebral infarction.

In the present study, we did not focus on the duration of hypotension. With the strict perioperative anesthesia management, the incidence of hypotension during the surgery of MMD remained low. Besides, our anesthesia management emphasized the stability of perioperative hemodynamics. Intraoperative hypotension is less detrimental than blood pressure instability to MMD patients (13). Chronic mild cerebral ischemia allows a tolerance to stable hypotension, whereas unstable blood pressure fluctuations, particularly rapid and significant drops, increase the risk of ischemic stroke.

Given that the steno-occlusive pathology mainly involves the terminal portions of bilateral hemispheres, hence, ischemic complications following revascularization surgery for MMD are not isolated to the ipsilateral hemisphere (29). However, the precise mechanism underlying acute contralateral cerebral infarctions has not been clearly defined and was regarded as multifactorial. A relevant study, conducted by Kim et al. (30) found that the potential mechanism for contralateral stroke was the redistribution of intracranial blood away from the contralateral side after revascularization. Specifically, the cerebral blood flow of the ipsilateral side was restored by the revascularization, and this might result in a reduction of the contralateral collateral flow. In addition, Parray et al. (31) indicated that the hemodynamic interrelationship between the two hemispheres and potential connections of bi-hemispheric vascular networks might be another potential mechanism. Though we have investigated the risk factors for POCI in adults MMD, our study did not differentiate between ipsilateral and contralateral cerebral infarctions. Besides, as outlined in prior work, contralateral cerebral infarctions associated with MMD may occur less frequently than ipsilateral cerebral infarctions (22, 29, 32). Considering different incidence and mechanism among ipsilateral and contralateral cerebral infarctions, further studies are warranted to identify their respective risk factors.

## 5 Advantages and limitations

Our study presented several advantages. First, we thoroughly analyzed clinical data in multiple aspects, including the baseline characteristics, demographic data, imaging findings, laboratory testing, surgery-related variables, and anesthesia-related variables. Second, univariable and multivariable logistic regression with stepwise backward greatly prevented overfitting of the nomogram. Third, a large sample size elevated the reliability. However, limitations should also be concerned. First, this is a retrospective analysis based on case-system data, leading to potential recall and reporting biases. Second, the application of our nomogram to pediatric MMD patients requires a further exploration. Furthermore, our study did not differentiate between ipsilateral and contralateral cerebral infarctions. Identifying new cerebral infarctions at different anatomical sites and on different operative sides may enable more precise management of patients with MMD. Lastly, with the advancements in machine learning technology, emerging feature selection methods could be integrated into our study to enhance the performance of the prediction model.

## 6 Conclusion

We constructed a nomogram, incorporating preoperative cerebral infarction, PCA anomalies, PTAD, pre-anesthesia MAP>102.83 mmHg, TOT>212.50 min, preoperative FIB>2.75 g/L, and MCHC, to effectively predict POCI after revascularization of adult MMD. The nomogram is validated to present acceptable performances of discrimination, calibration, and clinical applicability.

## Data Availability

The raw data supporting the conclusions of this article will be made available by the authors, without undue reservation.

## Glossary

95% CI: 95% confidence interval
A/G: albumin-to-globulin ratio
ACA: anterior cerebral arteries
Alb: albumin
ALP: alkaline phosphatase
APTT: activated partial thromboplastin time
AUC: area under the curve
CBC: complete blood count
CRP: C-reactive protein
CT: computerized Tomography
DBIL: direct bilirubin
DBP: diastolic blood pressure
DCA: decision curve analysis
EMS: Encephalo-Myo-Synangiosis
FBG: fasting blood glucose
FC: free cholesterol
FIB: fibrinogen
HB: hemoglobin
HDL: high-density lipoprotein
LDH: lactate dehydrogenase
LDL: low-density lipoprotein
MAP: mean arterial pressure
MCA: middle cerebral artery
MCHC: mean corpuscular hemoglobin concentration
MMD: moyamoya disease
MRI: Magnetic Resonance Imaging
NLR: neutrophil-to-lymphocyte ratio
OR: odds ratio
PCA: posterior cerebral artery
PLT: platelet count
POCI: postoperative new-onset cerebral infarction
PT: prothrombin time
PTAD: post-transit arterial development
P_V_CO_2_: venous blood carbon dioxide
RDW: red cell distribution width
ROC: operating characteristic curve
SBP: systolic blood pressure
SD: standard deviation
STA: superficial temporal artery
TAT: total anesthesia time
TBIL: total bilirubin
TC: total cholesterol
TG: triglyceride
TOT: total operative time
TP: total protein
