# Supplementary material for: A nomogram to predict postoperative new-onset cerebral infarction after revascularization of moyamoya disease in adults and its validation: a retrospective study
Source: Front Neurol. 2025 Jan 24;16:1537755. doi: 10.3389/fneur.2025.1537755 (PMC11876968; doi:10.3389/fneur.2025.1537755)
Supplement: Supplementary file 1 [file Table_1.DOCX]

Supplementary Table 1: Detailed protocols during the anesthesia management.

| Procedure | Specific details |
| --- | --- |
| Routine anesthetic procedures | Preparation for Anesthesia: Patients were instructed to fast for 8 hours and abstain from drinking for 4 hours prior to surgery. Upon arrival at the operating room, venous access was established. We routinely employed a Philips multifunctional monitor to continuously monitor heart rate (HR), oxygen saturation (SpO2), blood pressure (BP), and electrocardiogram (ECG). Under local anesthesia, radial artery puncture catheterization was performed to monitor invasive arterial blood pressure. |
| Anesthesia induction protocol | Administration and Mechanical Ventilation: Midazolam (0.05 mg/kg), etomidate (0.3 mg/kg), fentanyl (5 μg/kg), and vecuronium bromide (0.15 mg/kg) were administered intravenously. Endotracheal intubation was performed after the onset of the drug effects. The patient was then connected to the anesthesia machine for mechanical ventilation, with the tidal volume set at 8-10 ml/kg, a respiratory rate of 12 breaths/min, and an inspiratory/expiratory (I/E) ratio of 1:2. Subsequently, central venous pressure (CVP) was monitored via deep vein puncture based on the different sides of minimally invasive surgical techniques (MMD): right internal jugular vein puncture and cannulation were performed for the right side, while femoral vein puncture and cannulation were utilized for the left side to prevent displacement of the deep venous catheter due to head position changes. |
| Anesthesia maintenance protocol | Micropump infusion: Propofol 4-10 mg/kg^-1^/h^-1^  Remifentanil 0.5-1.0μg/kg^-1^/min^-1^  cis-Atracurium 0.2 mg/kg^-1^/h^-1^  Dexmedetomidine 0.2 μg.kg^-1^.h^-1^. |
| Blood Pressure Management | Systolic blood pressure (SBP) was maintained at 130-140 mmHg without hypertension and 140-150 mmHg in the presence of hypertension, with continuous administration of vasoactive drugs such as norepinephrine and phenylephrine as needed. |
| Carbon Dioxide Management | Partial pressure of carbon dioxide (PaCO2) was maintained at 38-42 mmHg following the induction of anesthesia until the patient was stable. Arterial blood gases were measured prior to skin incision, and the difference between PaCO2 and the partial pressure of end-expiratory carbon dioxide (PetCO2) was analyzed. This information was used to maintain the target PaCO2 by adjusting tidal volume and respiratory rate while ensuring the stability of the internal environment. |
| Other aspects of control | Body temperature was maintained above 36°C. Fentanyl was administered at a dose of 3 μg/kg prior to skin incision and again 30 minutes before the end of surgery.  Volume Control: Central venous pressure (CVP) was maintained at 6-12 cm H2O, with a pulse pressure variation (PPV) of less than 3%. Urine output was monitored in real-time to ensure appropriate fluid management. |
| Postoperative Care | Anesthetic maintenance drugs were discontinued at the end of the surgery. Upon completion of the procedure, all patients were transferred to the post-anesthesia care unit (PACU) or neonatal intensive care unit (NICU) with an endotracheal tube in place, where they received ventilator-assisted breathing and continuous monitoring of vital signs, including invasive blood pressure maintained at 120-130 mmHg. |
| Postoperative Assessment and Management | Upon awakening, muscle strength was assessed. If the patient exhibited significant pain agitation, an additional dose of fentanyl (0.1 mg) was administered, along with a single dose of propofol (0.5 mg/kg) until the patient was fully alert. Arterial blood gases were measured, confirming an oxygenation index greater than 200 mmHg and the absence of carbon dioxide accumulation. The endotracheal tube was removed once the internal environment was stable. |

Supplementary Table 2: Transformation of continuous variables into categorical variable truncations

| Variables | Category of variables | Detail Category | Methods for selecting thresholds | Variable Explanation |
| --- | --- | --- | --- | --- |
| BMI | multicategory | 1 Low-weight  2 Normal-weight  3 Overweight  4 Obese | BMI stratification | 1 = “BMI < 18.5kg/m2”;  2 = “18.5-24.9kg/m2”  3 = “25-29.9 kg/m2”  4 = “BMI > 30 kg/m2” |
| AGE | multicategory | 1 young people  2 middle-aged people  3 middle-aged and elderly | Age stratification | 1=“18-39 year”  2=“40-59 year”  3=“60-65year” |
| High SBP values | bicategory | 1 Yes  0 No | ROC-curve method | 1=“≥140mmHg”  0=“<140mmHg” |
| High DBP values | bicategory | 1 Yes  0 No | ROC-curve method | 1=“≥85.5mmHg”  0=“<85.5mmHg” |
| High fasting blood glucose | bicategory | 1 Yes  0 No | physiological threshold  (N:3.9-6.1mmol/L) | 1=“≥6.1 mmol/L”  0=“<6.1 mmol/L” |
| Hypercholesterolemia | bicategory | 1 Yes  0 No | physiological threshold  (N:3-5.7 mmol/L) | 1=“≥5.7 mmol/L”  0=“<5.7 mmol/L” |
| Low HDL | bicategory | 1 Yes  0 No | physiological threshold  (N:0.94-2 mmol/L) | 1=“<0.94mmol/L”  0=“≥0.94 mmol/L” |
| High LDL | bicategory | 1 Yes  0 No | physiological threshold  (N:1.89-3.1mmol/L) | 1=“≥3.1 mmol/L”  0=“<3.1 mmol/L” |
| hypertriglyceridemia | bicategory | 1 Yes  0 No | physiological threshold  (N:≤0.7mmol/L) | 1=“≥0.7 mmol/L”  0=“<0.7 mmol/L” |
| hyperbilirubinemia | bicategory | 1 Yes  0 No | physiological threshold  (N≤26μmol/L) | 1=“≥26 μmol/L ”  0=“<26μmol/L ” |
| PLT low value | bicategory | 1 Yes  0 No | physiological threshold  (N:125-350*10^9^/L) | 1=“<125*10^9^/L ”  0=“≥125*10^9^/L” |
| Albumin Level | bicategory | 1 Yes  0 No | physiological threshold  (N:40-55 g/L) | 1=“<40 g/L ”  0=“≥40 g/L ” |
| anemia | bicategory | 1 Yes  0 No | physiological threshold  （N:130-175 g/L） | 1=“<130 g/L ”  0=“≥130 g/L” |
| Volume width of erythrocyte distribution | bicategory | 1 Yes  0 No | physiological threshold  (N:0-14%) | 1=“≥14%”  0=“<14%” |
| Total Anesthesia Time | bicategory | 1 Yes  0 No | ROC-curve method | 1=“≥245min”  0=“<245 min” |
| Total Operation Time | bicategory | 1 Yes  0 No | ROC-curve method | 1=“≥212.5 min ”  0=“<212.5min” |
| Venous blood carbon dioxide level（P_V_CO_2_） | bicategory | 1 Yes  0 No | ROC-curve method | 1=“≥24.65mmol/L ”  0=“<24.65mmol/L” |
| Fibrinogen (FIB) | bicategory | 1 Yes  0 No | ROC-curve method | 1=“≥2.75g/L”  0=“<2.75g/L” |
| Mean Arterial Pressure(MAP) | bicategory | 1 Yes  0 No | ROC-curve method | 1=“≥102.83mmHg”  0=“<102.83mmHg” |

Supplementary Table 3:

Results of Univariate analysis of other indicators.

|  | | Univariate analysis | |
| --- | --- | --- | --- |
| Variables | OR(95% CI) | | P value |
| Acceptor Vessel |  | |  |
| No revascularization | Reference | |  |
| STA-AB | 1.715(0.444-6.620) | | 0.434 |
| STA-PB | 1.887(0.553-6.440) | | 0.311 |
| STA- PaB | 3.053(0.788-11.826) | | 0.106 |
| STA-FB | 9.667(0.474-197.279) | | 0.140 |
| ESM |  | |  |
| No | Reference | |  |
| Yes | 0.714(0.359-1.421) | | 0.337 |
| Percentage of neutrophils(%) | 0.994(0.965-1.024) | | 0.700 |
| Percentage of lymphocytes(%) | 1.006(0.975-1.038) | | 0.704 |
| Percentage of monocytes(%) | 1.006(0.871-1.161) | | 0.939 |
| Percentage of eosinophils(%) | 1.056(0.904-1.234) | | 0.490 |
| Percentage of basophils(%) | 0.686(0.262-1.795) | | 0.442 |
| Alanine aminotransferase(ALT, U/L) | 1.005(0.993-1.016) | | 0.420 |
| Aspartate aminotransferase(AST, U/L) | 1.009(0.984-1.035) | | 0.470 |
| Glutaminotransferase(GGT, U/L) | 1.005(0.997-1.013) | | 0.216 |
| Choline esterase (ChE, KU/L) | 1.027(0.888-1.188) | | 0.717 |
| TG/HDL | 0.979(0.816-1.174) | | 0.821 |
| Apolipoprotein AⅠ(g/L) | 0.773(0.201-2.976) | | 0.708 |
| Apolipoprotein B(g/L) | 0.412(0.126-1.349) | | 0.143 |
| CRP/PL | 0.619(0.134-3.426) | | 0.639 |
| Hyperbilirubinemia |  | |  |
| No | Reference | |  |
| Yes | 0.932(0.546-1.592) | | 0.798 |
| LDL |  | |  |
| <0.94 mmol/L | Reference | |  |
| ≥0.94 mmol/L | 0.565（0.247-1.291） | | 0.176 |
| HDL |  | |  |
| ≥3.1 mmol/L | Reference | |  |
| <3.1 mmol/L | 1.284(0.768-2.147) | | 0.340 |

Supplementary Table 4:

Scores for each indicator of the Nomogram.

| Variables | Points |
| --- | --- |
| Fib Points |  |
| Fibrinogen<2.75g/L | 73 |
| (Fibrinogen≥2.75g/L) | 80 |
| PTAD(Post-transit arterial development) Points |  |
| No | 73 |
| Yes | 80 |
| MCHC Points |  |
| 1(0) | 4 |
| 2(50) | 7 |
| 3(100) | 19 |
| 4(150) | 30 |
| 5(200) | 42 |
| 6(250) | 54 |
| 7(300) | 65 |
| 8(350) | 77 |
| 9(400) | 88 |
| 10(450) | 100 |
| MAP Points |  |
| MAP<102.83mmHg | 73 |
| MAP≥102.83mmHg | 87 |
| Total Operation Time Points |  |
| Operation Time≤212.5min | 73 |
| Operation Time>212.5min | 82 |
| PCA Points |  |
| No | 73 |
| Yes | 82 |
| CI history Points |  |
| CI history 1 | 73 |
| CI history 2 | 84 |

P r( )

1 440 3.862e-05

2 460 2.256e-04

3 480 1.316e-03

4 500 7.640e-03

5 520 4.304e-02

6 540 2.081e-01

7 560 6.055e-01

8 580 8.997e-01
